# MicroRNA 133A Regulates Cell Proliferation, Cell Migration, and Apoptosis in Colorectal Cancer by Suppressing CDH3 Expression

**DOI:** 10.7150/jca.82916

**Published:** 2023-04-09

**Authors:** Grinsun Sharma, Ji-Su Mo, Santosh Lamichhane, Soo-Cheon Chae

**Affiliations:** 1 Department of Pathology, School of Medicine, Wonkwang University, Iksan, Chonbuk 54538, Republic of Korea; 2 Digestive Disease Research Institute, Wonkwang University, Iksan, Chonbuk 54538, Republic of Korea

**Keywords:** MIR133A, CDH3, Cell viability, Colorectal Cancer, EMT

## Abstract

MicroRNAs are endogenous, non-coding RNA that play an essential role in colorectal carcinoma (CRC) pathogenesis by targeting specific genes. This research aimed to determine and validate the target genes of the *MIR133A* associated with CRC. We verified that cadherin 3 (*CDH3*) is the direct target gene of *MIR133A* using a luciferase reporter assay, quantitative RT-PCR, and western blot analyses. *CDH3* mRNA and protein expression were reduced significantly in CRC cells after transfection with *MIR133A* or *siCDH3*. We also verified that *MIR133A* regulated CDH3-mediated catenin, matrix metalloproteinase, apoptosis, and the epithelial-mesenchymal transition (EMT) pathway. Knockdown of *CDH3* in CRC cell lines by *siCDH3* produced similar results. Compared with adjacent non-tumor tissues, CDH3 protein expression was upregulated in CRC tissues, which is further confirmed by immunohistochemistry. Additionally, molecular and functional studies revealed that cell viability, migration, and colony formation were significantly reduced, and apoptosis was increased in CRC cell lines transfected with *MIR133A* or *siCDH3*. Our results suggest that *MIR133A* regulates *CDH3* expression in human CRC.

## Introduction

Colorectal carcinoma (CRC) is a common cancer of the digestive tract with a high death rate 1. In 2020, about 19.3 million novel cases and 10 million cancer-related mortalities were recorded worldwide. CRC comprises about 10% of new cases and 9.4% of cancer-related deaths, which are increasing globally 2. Several studies have shown a global prevalence of CRC of about 4-5%. CRC risk factor comprises age, inflammatory bowel disease (IBD) history, sedentary lifestyle, obesity, diet, smoking, and alcohol 3. In many developing countries, high incidence and poor detection of CRC have increased the need for early diagnosis and treatment. Hence, different prognostic biomarkers, including treatments for CRC, should be explored.

Cadherins are cell adhesion molecules that help cells to adhere to each other and help cells migrate via epithelial-mesenchymal transition (EMT) 4,5. They control calcium-dependent cellular adhesion and bind to various cell types and the extracellular matrix (ECM), maintaining cellular structure. Cadherin 3 (*CDH3*, also known as *PCAD*) has a molecular weight of 118kDa; comprises extracellular, transmembrane, and cytoplasmic domains; and promotes homotypic interactions. In normal cells, CDH3 regulates cell growth and migration, but CDH3 may have opposing effects in tumors depending on type: CDH3 promotes tumorigenesis in pancreatic, breast, and gastric cancer but has the opposite effect in non-small cell lung carcinoma, liver cancer, and melanoma 6,7. A previous study reported that CDH3 is upregulated in colon adenocarcinoma, and patients with high CDH3 have a good prognosis for cancer 8,9. The aberrant expression of CDH3 in the colonic mucosa of CRC is the effect of abnormal gene upregulation through *CDH3* gene promoter hypomethylation 10,11.

MicroRNAs (miRNAs) are single-stranded, non-coding RNA that are expressed endogenously and attach to the target mRNA's 3′ untranslated regions (3′ UTR) and regulate gene expression by mRNA degradation or translational inhibition. Normal expression of miRNAs is tissue-specific and regulates various pathways involved in growth, cell differentiation, migration, and apoptosis, whereas aberrant expression of miRNAs leads to the development of human cancer 12. While miRNAs play a crucial role in cancer biology, the molecular mechanism underlying miRNA expression in cancer is not well understood. Many studies have demonstrated associations of miRNAs, including microRNA 133A (*MIR133A*, also known as *has-miR-133A*), with colon cancer pathogenesis 13,14,15. In our earlier research, we reported that *MIR133A* expression was reduced in CRC, which correlates with poor diagnosis in CRC patients 16,17. The *CDH3* gene was identified as one of the candidate targets of *MIR133A* in our previous study using *MIR133A1* knock-in cells and was confirmed using TargetScan, miRanda, and miRWalk algorithms 16.

Here, we reported *CDH3* as a direct target gene of *MIR133A*. We showed the association of *MIR133A* and *CDH3* via human CRC cell lines and tissues. We have also used MTT, colony formation, migration assays, and flow cytometry to further verify the correlation. Our results suggest that *MIR133A* regulates *CDH3* in human colorectal cancer and may be helpful for CRC.

## Materials and Methods

### Patient sample

This study used tissue samples from the Biobank of Wonkwang University Hospital. With Institutional Review Board permission and informed consent from participants (WKIRB-202006-BR-023), we received four CRC tissue and four adjacent normal samples from colon cancer patients [2 TNM stage 3 (1 female and 1 male) and 2 TNM stage 4 (1 female and 1 male)]. The mean ages of the colon cancer patients were 60. These samples were used to examine protein expression using western blotting and *in situ* CDH3 expression using immunohistochemistry.

### Cell culture

Human CRC SW480, Caco2, and LoVo cell lines were acquired from Korean Cell Line Bank (KCLB, Seoul, Korea) or the American Type Culture Collection (ATCC). The cells were cultured in RPMI-1640 (Hyclone, UTAH, USA) or MEM Alpha (Hyclone, UTAH, USA) supplemented with 10% fetal bovine serum (FBS) in 5% CO_2_ at 37⁰C in a humidified atmosphere.

### RNA extraction and quantitative real-time PCR

The RNA extraction and quantitative RT-PCR (qRT-PCR) were done as our previous study 18,19. Each experiment was analyzed three times. The primers that were used are in Supplementary Table 1.

### Transfection of oligonucleotides

The SW480 and Caco2 cells were cultured in RPMI and MEM Alpha medium. *MIR133A* mimic (*hsa-MIR-133A*, pre-miR miRNA precursor AM17100, product ID: PM12946) and negative control (Ambion, USA) were added at 50 nmol/mL for transfection, which was performed as previously described 20,21. Lipofectamine RNAiMAX (Invitrogen) and siPORT NeoFX (Ambion) were used as transfecting agents. The *CDH3* small interfering RNA (siRNA)(Sigma-Aldrich) and negative control siRNA (Ambion) were used according to the manufacturer's protocols. After transfection, cells were cultured for 72 hours for protein analyses.

### Plasmid constructs and luciferase assay

Wild-type (WT) or mutant-type (MT) constructs of *CDH3* 3′ UTR were created using PCR primers as indicated in Supplementary Table 1. Then, constructs were ligated into the pmirGLO™ Dual-Luciferase miRNA target expression vector (Promega, USA). All constructs were confirmed by DNA sequencing. In this assay, 5 × 10^4^ SW480 cells/well were seeded into 24-well plates and transfected with WT-*CDH3* or MT-*CDH3* constructs and with *MIR133A* or *MIRNC* (negative control) using lipofectamine (Invitrogen) and siPORT^TM^ NeoFX^TM^ transfecting agents (Ambion). The results were evaluated as previously described 22,23. All experiments were repeated three times.

### Western blot analyses

The SW480, Caco2, and LoVo cells (2 × 10^5^ cells/well) were cultured in cell culture plates for 72 hours after transfection, followed by lysis with RIPA buffer, and lysates were centrifuged at 12000 RPM (4⁰C for 15 min). Protein concentration was calculated using BSA reagents (Thermo Fisher, USA). An equal amount of cell protein was boiled in Laemmli buffer and subjected to SDS-PAGE, followed by transfer to a PVDF membrane (Millipore Corporation, USA). The PVDF membranes were incubated with 3% BSA (BOVOGEN Biologicals, Australia) in 1× PBS with 1% Tween^®^ 20 (iNtRON Biotechnology) for one hour at RT. Membranes were incubated overnight at 4⁰C with primary anti-CDH1 (3195S, 1:1000), -VIM (5741S, 1:1000), -CTNNB1 (610153, 1:2000), -GSK3B (NBP1-47470, 1:1000), -MMP1 (sc-373908, 1:500), -MMP2 (sc-13595, 1:500), -BCL2 (sc-7382, 1:500), -GAPDH (sc-47724, 1:2500), -CDH3 (sc74545, 1:500), -BIRC5 (sc-17779,1:500), -CTNND1 (sc-23873, 1:500), -CDH2 (sc-8424, 1:500), ‍‍‑CASP3(#9661, 1:1000), -CASP9 (#9502S, 1:1000), -CASP8 (#9746L, 1:1000), -RIPK3 (sc-374639, 1:500), and -MLKL (sc-293201, 1:500) antibodies and washed with PBST three times for 8 min/wash. After washing, membranes were incubated for one hour at RT with a secondary antibody (goat anti-rabbit, sc-2004, 1:5000, goat anti-mouse antibody 1:10000). Proteins were evaluated using ECL solution (Millipore) and FluorChem™-E System (Cell Biosciences, USA). For reblotting, a stripping buffer (Thermo Fisher, USA) was used. GAPDH was used as the control. Quantification was performed using ImageJ software. Each experiment was analyzed three times.

### MTT assay of cell viability

The SW480 cell line (2 × 10^4^) was used to assess cell viability. Transfection of SW480 cell line was done with 50 nM *MIR133A* or *MIRNC* in a 96-well plate. The MTT reagent (Sigma-Aldrich, USA) was dissolved at a concentration of 0.5 mg/ml in RPMI medium. After removing the RPMI media, MTT solution was added in every well. After covering in aluminum foil, cell plates were placed in 37⁰C incubator for three hours. After removing the media, DMSO (Duchefa Biochemie, Netherlands) was used to dissolve MTT crystals. The plates were then placed in a shaker for 30 min, and optical density was measured at 560 nm using a GloMax^®^-Multi+ Detection System (Promega). Each experiment was analyzed three times.

### Scratch wound-healing assay

A monolayer of SW480 cells cultured in a 6-well plate was scratched to form a wound using a sterilized 200 µm pipette tip. Scratching was performed after 24 hours of transfection, and serum-free media was used to wash the wells to remove cell debris. The plates were then incubated for 72 hours. Representative photographs were obtained at 0, 24, 48, and 72 hours using a Canon DS-126431 camera system mounted in a phase-contrast Nikon TS100 microscope (10×). Each experiment was analyzed three times.

### Colony-forming assay

The SW480 cells (1000 cells/well) were transfected with *MIR133A* (50nM) or *MIRNC* in a 6-well plate and cultured in an incubator with 5% CO_2_ at 37⁰ C for 15-20 days for the formation of colonies. Media was changed every 72 hours, and cells were washed with 1X PBS. Colonies were stained by using 0.5 ml of 6% glutaraldehyde and 0.5% crystal violet, followed by incubation at RT for 30 minutes and staining solution was discarded, and the cells were cleansed with distilled water and counted after drying in air. Each experiment was analyzed three times.

### Immunohistochemistry (IHC)

Human colon sections were formalin-fixed, implanted in paraffin, and cut for IHC reaction. The expression of CDH3 was evaluated as described previously 21,24.

### Apoptosis analysis (flow cytometry)

The SW480 (2 × 10^5^ cells/well) were transfected with *MIR133A* (50nM) or *MIRNC* in 6-well plates for 72 hours. An Annexin V-FITC Apoptosis Detection Kit (Sigma-Aldrich, St. Louis, MO, USA) was used for flow cytometry as in our previous experiment 18,24.

### Statistical analyses

Each experiment was analyzed three times. The results are reported as mean ± standard deviation. Microsoft Excel and GraphPad Prism 8 were used to complete all statistical analyses. Two-tailed Student's t-tests were used for comparison, and *P* values < 0.05 were considered significant.

## Results

### *CDH3* is a direct target of *MIR133A*

**Figure 1:**
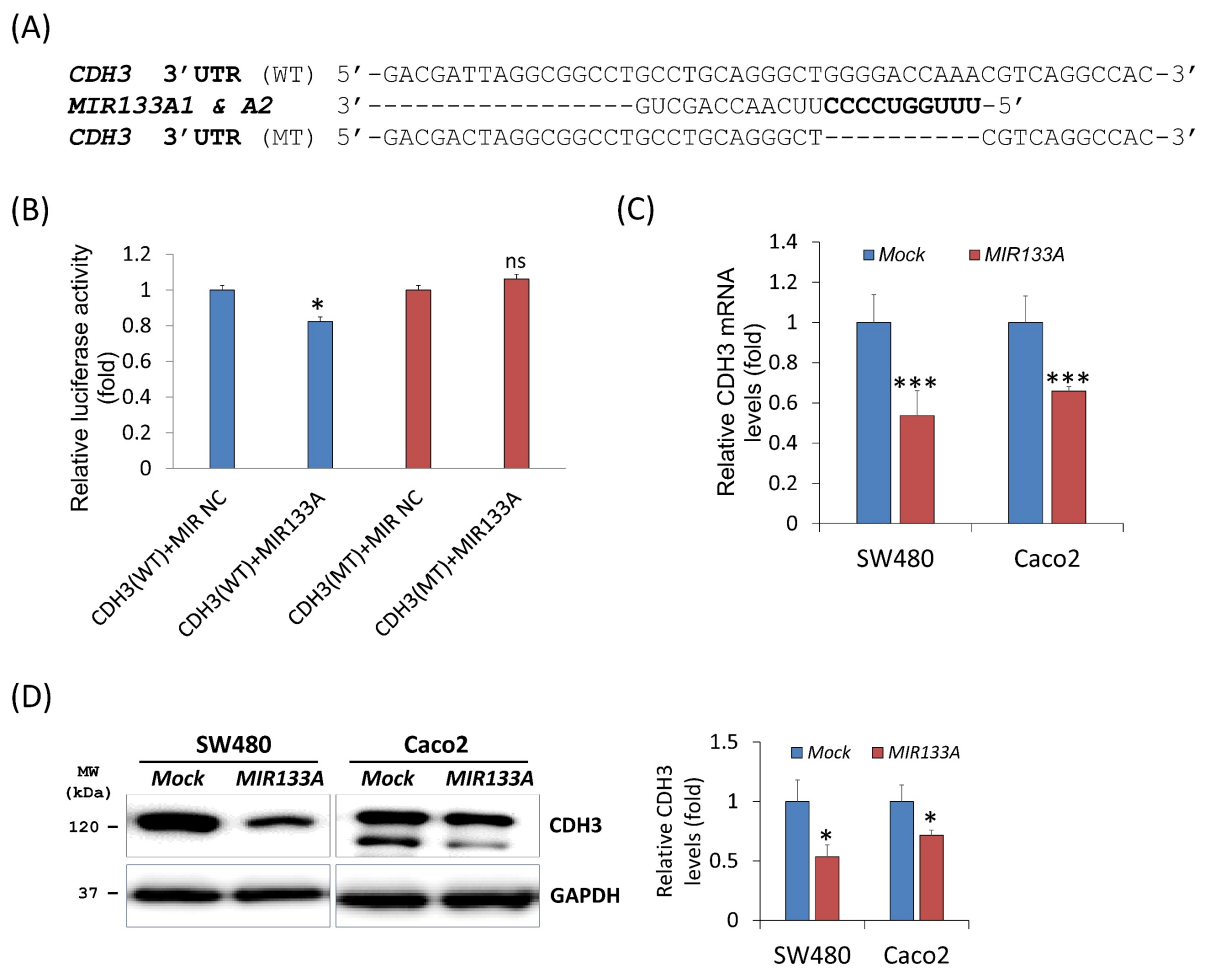
*CDH3* is a direct target of *MIR133A* (A) Sequence constructs of wild-type (WT) and mutant (MT) *MIR133A* target sites in the 3′ UTR of *CDH3*. A human *CDH3* 3′ UTR containing the WT and MT *MIR133A* binding constructs was cloned to a luciferase vector. (B) A luciferase reporter plasmid with WT or MT *CDH3* 3′ UTR was co-transfected into SW480 cells. Luciferase activity was assayed using a dual-luciferase system. Results are shown as the relative firefly luciferase activity normalized to that of renilla luciferase. (C) qRT-PCR analyses of *CDH3* mRNA expression in SW480 and Caco2 cells after transfection with *MIRNC* or *MIR133A*. Following transfection, cells were incubated for 48 hour. Values are presented as fold change with respect to mock-transfected controls. (D) Western blot analyses of cellular CDH3 protein in SW480 and Caco2 cells after transfection with *MIRNC* or *MIR133A*. Following transfection, cells were incubated for 72 hours, and proteins were collected. Data were obtained from three independent experiments, and *p* values were calculated using t test (ns = not significant, * *P* < 0.05, *** *P* < 0.001).

To evaluate the influence of *MIR133A* on the* CDH3* 3′ UTR region, we construct a wild-type (WT) *CDH3* 3′ UTR region (predicted to interact with *MIR133A*) into a luciferase vector (Fig. 1A). Compared with the negative control, luciferase activity was significantly reduced in the *MIR133A*-transfected cells (Fig. 1B). Additionally, a mutant-type (MT) *CDH3* 3′ UTR was constructed by deleting the seed sequence of *MIR133A*; however, *MIR133A*-mediated luciferase suppression was not present at the mutant-transfected cells (Fig. 1A, B). Furthermore, qRT-PCR was used to assess *CDH3* mRNA expression in *MIR133A*-transfected cells. With respect to control cells, *CDH3* mRNA expression was significantly reduced in *MIR133A*-transfected cells (Fig. 1C). Likewise, CDH3 protein expression was significantly decreased in the *MIR133A*-transfected group (Fig. 1D). These findings demonstrate that *MIR133A* directly targets CDH3.

### *MIR133A* modulates the *CDH3*-mediated EMT pathway

**Figure 2:**
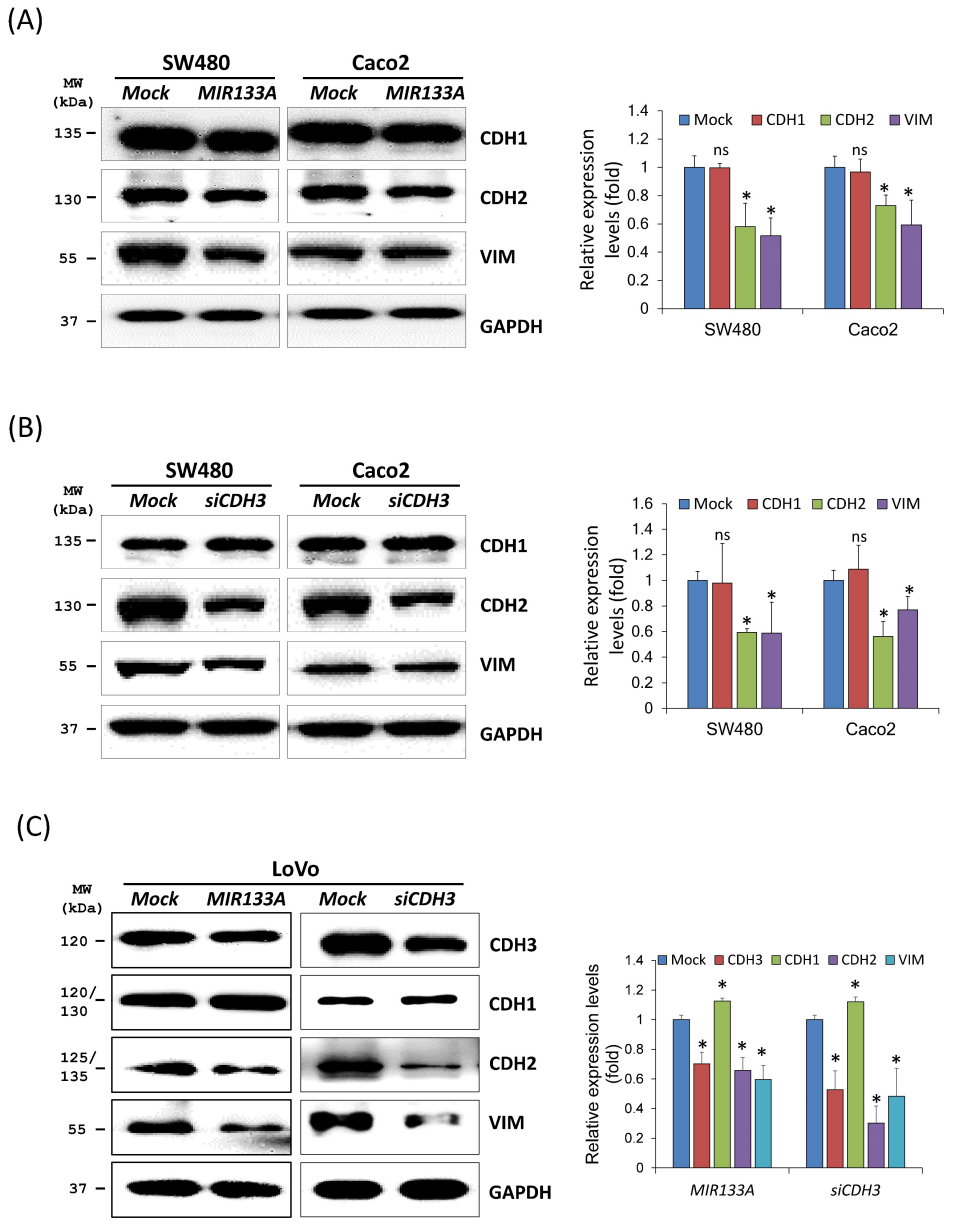
*MIR133A* modulates the *CDH3*-mediated EMT pathway Western blot analyses of CDH1, CDH2, and VIM protein expression in SW480 and Caco2 cell lines after transfection with *MIR133A* (A) or *siCDH3* (B). (C) Western blot analyses of CDH3, CDH1, CDH2, and VIM protein expression in LoVo cell lines after transfection with *MIR133A* or si*CDH3*. Data were obtained from three independent experiments, and *p* values were calculated using t test (ns = not significant, * *P* < 0.05).

The EMT represents a complex program in which epithelial cells lose their cell identity and attain a mesenchymal phenotype. EMT is also active in cancer cells, leading to apoptosis resistance, tumor metastasis, and the acquisition of cancer stem cell characteristics 25. As CDH3 has been reported to affect cell migration and invasion 26, we aimed to verify the association between reduction in *CDH3* expression mediated by *MIR133A* and induction of the EMT. Downstream proteins of the EMT pathway in SW480, Caco2, and LoVo cell lines were measured. Western blotting results showed that cadherin 2 (CDH2, also known as *N-*cadherin) and vimentin (VIM) expression was significantly reduced after *MIR133A* transfection in CRC cell lines (Fig. 2A). Additionally, *CDH3* gene silencing revealed that expression of CDH2 and VIM proteins was significantly reduced (Fig. 2B). The expression of CDH1 (also known as E-cadherin) was not significantly changed in both *MIR133A* and *siCDH3* overexpressed cell lines (Fig. 2A, B).

The ability to metastasize was more significant in the LoVo cell line, so we measured changes in CDH1, CDH2, and VIM expression using this line (Fig. 2C). Expression of both CDH2 and VIM was significantly downregulated by *MIR133A* and *siCDH3* transfection, whereas CDH1 expression was upregulated by *MIR133A* and *siCDH3* transfection (Fig. 2C). These data indicate that *MIR133A* regulates a *CDH3*-mediated EMT signaling pathway in CRC cells.

### *MIR133A* modulates the catenin pathway in CRC cell lines

**Figure 3:**
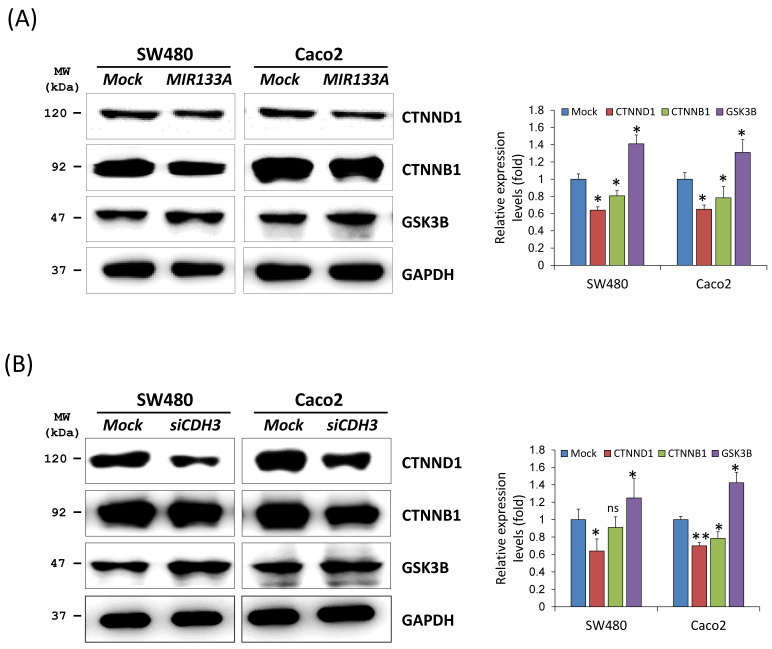
*MIR133A* modulates the catenin pathway in CRC cell lines (A) Western blot analyses of CTNND1, CTNNB1, and GSK3B protein expression in SW480 or Caco2 cell lines after transfection with *MIR133A.* (B) Western blot analyses of CTNND1, CTNNB1, and GSK3B protein expression in SW480 or Caco2 cell lines after transfection with *siCDH3*. Data were obtained from three independent experiments, and *p* values were calculated using t test (ns = not significant, * *P* < 0.05, ** *P* < 0.01).

Cadherin mediates cellular adhesion and tissue morphology in association with catenin beta 1 (CTNNB1) 8. Also, *CDH3* is strongly associated with cytoplasmic accumulation of catenin delta 1 (CTNND1, also known as CAS and p120) 27. To identify functional interactions of *MIR133A* within the catenin pathway, we carried out a western blot analysis using total cell isolates from SW480 and Caco2 cells. Our results showed that transfection of *MIR133A* in CRC cell lines significantly reduced CTNND1 and CTNNB1 protein expression in comparison to the mock (Fig. 3A). Additionally, *siCDH3* transfection of SW480 and Caco2 cell lines was used to study the interaction of *siCDH3* in the catenin pathway and indicated that protein expression of CTNND1and CTNNB1 was downregulated by *CDH3* gene silencing (Fig. 3B). In comparison, glycogen synthase kinase 3 beta (GSK3B) was increased after both transfections (Fig. 3A, B).

### *MIR133A* modulates the apoptosis pathway in CRC cell lines

**Figure 4:**
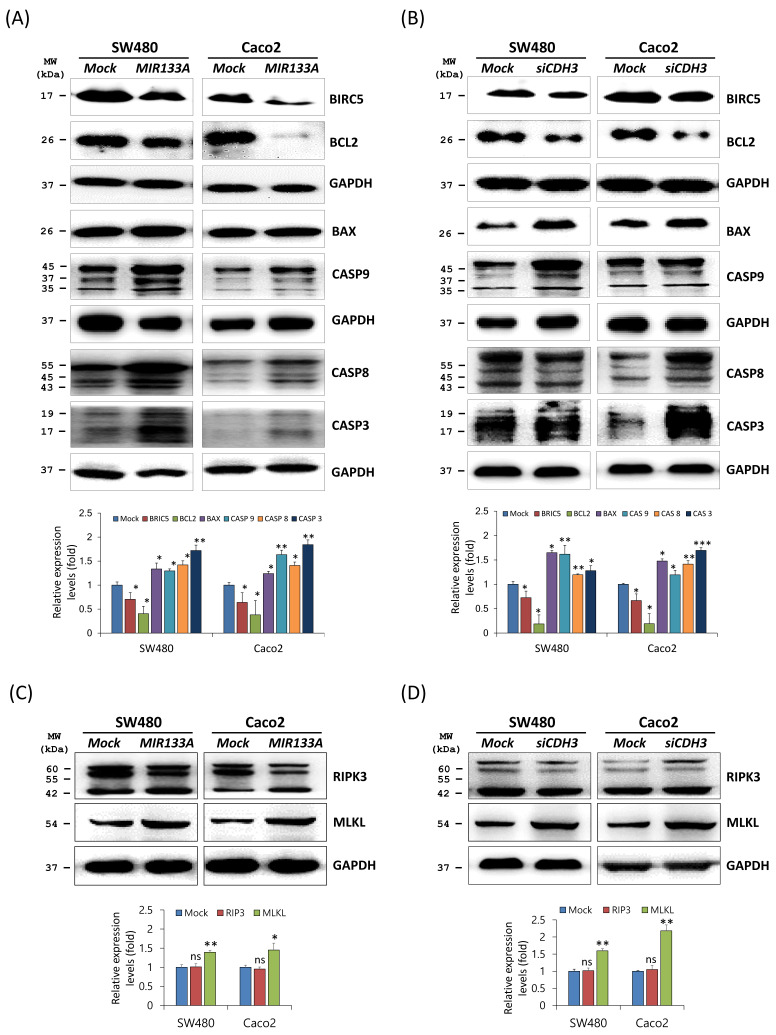
*MIR133A* modulates the apoptosis pathway in CRC cell lines (A) Western blot analyses of BIRC5, BCL2, BAX, CASP9, CASP8, and CASP3 protein expression in SW480 or Caco2 cell lines after transfection with *MIR133A*. (B) Western blot analyses of BIRC5, BCL2, BAX, CASP9, CASP8, and CASP3 protein expression in SW480 or Caco2 cell lines after transfection with *siCDH3.* (C, D) Western blot analyses of RIPK3 and MLKL protein expression after transfection with *MIR133A* or *siCDH3*. Data were obtained from three independent experiments, and *p* values were calculated using t test (ns = not significant, * *P* < 0.05, ** *P* < 0.01, *** *P* < 0.001).

Apoptosis is programmed cell death during normal cell development and is a homeostatic mechanism to maintain cells 28. To determine the functional interaction of *MIR133A* in the apoptosis pathway, western blot analyses was performed using SW480 and Caco2 cells. Expression of the BCL2 apoptosis regulator (BCL2) and the baculoviral IAP repeat-containing 5 (BIRC5, as known as API4 and survivin) was significantly reduced, whereas expression of the BCL2-associated X (BAX) apoptosis regulator, caspase 9 (CASP9), CASP8, and CASP3 was upregulated in *MIR133A* overexpressed SW480 and Caco2 cell lines (Fig. 4A). The data showed that BCL2 and BIRC5 were significantly downregulated and BAX, CASP9, CASP8, and CASP3 were increased by *CDH3* gene silencing (Fig. 4B). We also assessed changes in expression of the necroptosis marker receptor-interacting serine/threonine kinase 3 (RIP3) and mixed lineage kinase domain-like pseudokinase (MLKL). The expression of MLKL was upregulated by *MIR133A* and *siCDH3* transfection, whereas there was no change in RIP3 (Fig. 4C, D).

### *MIR133A* modulates the *CDH3*-mediated MMP1/MMP2 pathway of invasion and metastasis

**Figure 5:**
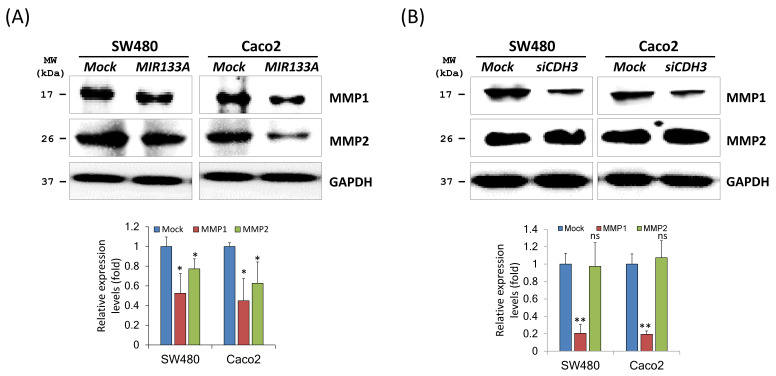
*MIR133A* modulates the *CDH3*-mediated MMP1/MMP2 pathway of invasion and metastasis (A) Western blot analyses of MMP1 and MMP2 protein expression in SW480 or Caco2 cell lines after transfection with *MIR133A* or mock transfection. (B) Western blot analyses of MMP1 and MMP2 protein expression in SW480 or Caco2 cell lines after transfection with *siCDH3*. Data were obtained from three independent experiments, and *p* values were calculated by t test (ns = not significant, * *P* < 0.05, ** *P* < 0.01).

Matrix metalloproteinases (MMPs) are endopeptidases that can degrade ECM components 29. In breast cancer, overexpression of *CDH3* will increase cell invasion and migration and increase MMP1 and MMP2 expression 30. To investigate the regulation of *MIR133A* in MMPs of CRC cells, western blot analyses of total cell isolates from SW480 and Caco2 cells were carried out. Expression of MMP1 and MMP2 was significantly reduced in MIR133A overexpressed SW480 and Caco2 cell lines (Fig. 5A). Additionally, *siCDH3* transfection revealed that MMP1 was reduced in CRC cell lines, whereas MMP2 expression was unchanged (Fig. 5B). These results indicate that CDH3 regulates only MMP1 expression in colorectal cancer.

### *MIR133A* inhibits CRC cell growth and cell cycle progression

**Figure 6:**
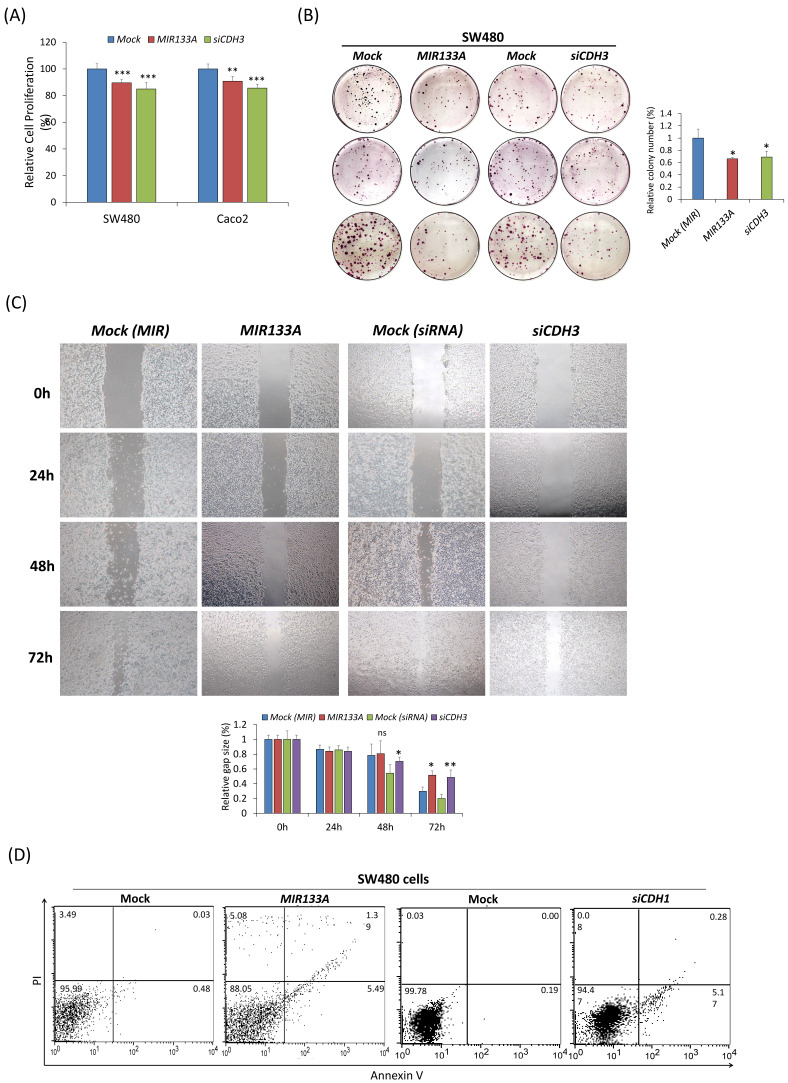
*MIR133A* inhibits CRC cell growth and cell cycle progression (A) MTT assay showed the reduction of cell viability of CRC cell lines after transfection with *MIR133A* or *siCDH3.* (B) Colony formation in SW480 cells was determined using a colony formation assay after transfection with *MIR133A* and *siCDH3.* (C) A scratch-wound-healing assay was performed in SW480 cells after transfection with *MIR133A* and *siCDH3*. Migration distance was measured at 0, 1, 2, and 3 days after wounding. (D) Flow cytometric analyses of apoptosis or necrosis in SW480 cells transfected with *MIR133A* and *siCDH3*. The number of each box indicates the percentage of annexin V-positive and PI-positive cells. Representative data from at least three independent experiments are shown. Each bar represents mean fold change above or below the control (± S.D). Differences were considered statistically significant compared with control (ns = not significant, * *P* < 0.05, ** *P* < 0.01, *** *P* < 0.001).

The functional analysis of *MIR133A* in SW480 and Caco2 cell lines was investigated using MTT, colony formation, cell migration, and flow cytometry assays. The MTT data confirmed that transfection of a *MIR133A* or *CDH3 siRNA* in SW480 and Caco2 cell lines significantly inhibited cell proliferation with respect to mock (Fig. 6A). The colony formation assay in the SW480 cell line indicated that transfection of *MIR133A* and si*CDH3* significantly reduced the number of colonies (Fig. 6B). These data indicate that MIR133A targets *CDH3* to suppress cell growth and colony formation in CRC cell lines.

Next, to study the effect of *MIR133A* on migration activity, SW480 cells were transfected with a *MIR133A* and *siCDH3*. The result indicated that migration of SW480 cells was reduced in *MIR133A*-transfected groups and was decreased significantly 72 hours post-transfection. Cells transfected with *CDH3 siRNA* showed similar inhibition of migration (Fig. 6C). These findings imply *MIR133A* regulates *CDH3* to limit migration.

Also, apoptosis data in figure 6D revealed that apoptosis and necrosis in SW480 cells were enhanced after *MIR133A* and *siCDH3* transfection compared with the mock (Fig. 6D).

### CDH3 expression in human CRC tissues

**Figure 7:**
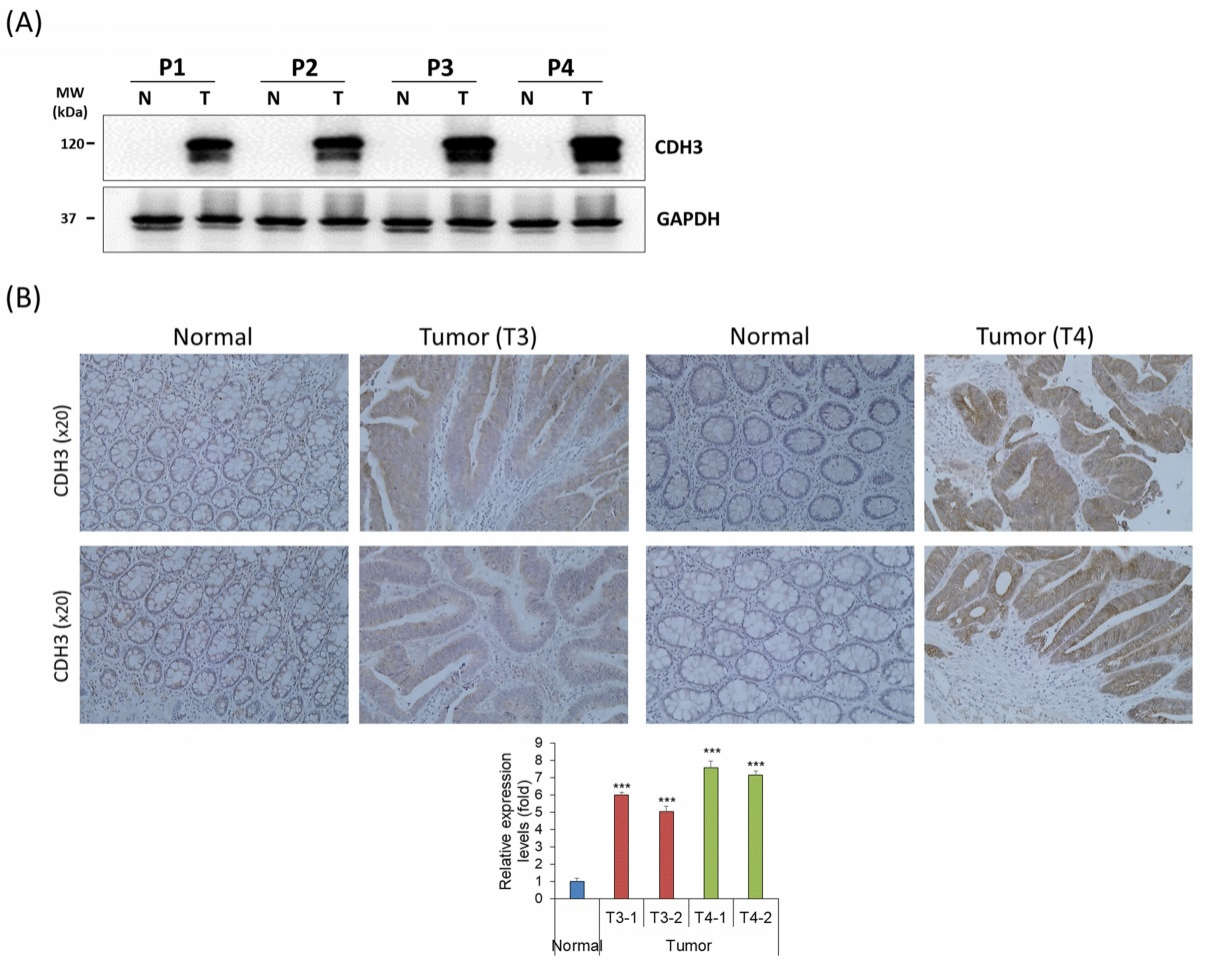
Endogenous CDH3 expression in human CRC tissues (A) CDH3 protein expression in paired human CRC and adjacent normal colon tissue samples from four patients (P#). P1 and P2 were tumor (T) stage 3 (T3), and P3 and P4 were T stage 4 (T4). (B) CDH3 IHC expression in paired colon cancer and adjacent normal tissue samples (N) (20× magnification) from four patients. Differences were considered statistically significant compared with control (*** *P* < 0.001).

CDH3 expression was analyzed in four human CRC tissue samples and adjacent colon tissues using western blot analysis and IHC. The CDH3 protein expression was significantly upregulated in all CRC tissues compared with the healthy normal colon tissues (Fig. 7A, B). The result displayed that CDH3 expression increased with tumor progression (Fig. 7A, B).

## Discussion

Colon cancer is the prominent cause of global cancer-associated mortality. Millions of new cases and thousands of cancer-related deaths are recorded each year. Activation of oncogenes and suppression of tumor suppressor genes are thought to lead to the initiation of CRC. An improved understanding of our knowledge of the oncogenes, mechanism of development, invasion, and reoccurrence of CRC, including validation of new biological markers of colon cancer, would aid in the early identification and diagnosis of colon cancer 31. Many studies have shown that miRNAs may have therapeutic significance as biomarkers as they regulate post-transcriptional gene expression in multicellular organisms. Our previous study showed that *MIR133A* is downregulated in CRC, which has also been reported in other studies 13,15,16, 32. However, the association of the *MIR133A* and *CDH3* genes in CRC is unexplored.

We identified CDH3 as the target gene of *MIR133A* (Fig. 1A, B) in CRC cells, and *CDH3* mRNA and protein expression was significantly reduced in *MIR133A* over-expressed cell lines (Fig. 1C, D). These data revealed that CDH3 expression is negatively linked with *MIR133A* in CRC tissues. Additionally, CDH3 protein expression was dramatically increased in CRC tissue in comparison with mock (Fig. 7A, B). Our data signified that the downregulation of *MIR133A* in CRC progression upregulates CDH3 expression in human CRC tissues.

After selecting the target gene and verifying mRNA and protein levels, we assessed the regulation of *MIR133A* in various signaling pathways involved in CRC tumorigenesis. The EMT converts static epithelial cells into an invasive mesenchymal phenotype. Downregulation of the *CDH1* gene and upregulation of *CDH3, CDH2*, and *VIM* in EMT is a hallmark of cancer metastasis 33. High expression of CDH3 disrupts epithelial adhesion and fosters the development of a more mesenchymal and progenitor-like phenotype, ultimately resulting in cancer 30. In this study, CDH3, VIM, and CDH2 were inhibited in *MIR133A* and *siCDH3* overexpressed cell lines (Fig. 2A, B, C), whereas there was no change in CDH1 expression (Fig. 2A, B). The extent of metastasis and degree of invasiveness of cancer cells are related to cancer prognosis. The ability to metastasize and MMP activities were found to be greater in the LoVo cell line than in SW480 cells 34,35. Hence, we assessed the regulation of the EMT pathway by *MIR133A* and *siCDH3* transfection in the LoVo cell line. The expression of CDH2 and VIM was downregulated, and CDH1 was upregulated by *MIR133A* and *siCDH3* transfection (Fig. 2C). These results suggest that *MIR133A* regulates the EMT pathway of colon cancer by regulating CDH3-dependent CDH2 and VIM expression. Similarly, MIR133A regulates the CRC progression and metastasis by targeting LIM and SH3 protein 1 and inhibiting the MAPK pathway 36.

Increased CDH3 expression also increases cell motility and migration in cancer by interfering with CTNND1 and CDH1. CDH3 binds to the juxtamembrane domain of CTNND1 and prevents the binding of CDH1, which stabilizes CTNND1 37. In this study, we investigated CTNND1 protein expression in a* MIR133A* overexpressed cell line. We found that CTNND1 expression is reduced after transfection with *MIR133A (Fig. 3A)*, and *siCDH3* overexpression produced similar results (Fig. 3B). The cadherin-catenin junction helps to maintain the cell structure and cell integrity and CTNNB1 helps in cell adhesion. A previous study reported that CTNNB1 is highly expressed in colorectal cancer 38. AKT phosphorylates CTNNB1 at Ser552, dissociating it from the WNT complex and leading to its accumulation in the cytosol and nucleus. *MIR133A* deactivates the PI3K/AKT pathway in CRC cell lines, and PI3K/AKT inhibits GSK3B and promotes cyclin D1 to increase its transcriptional activity, ultimately increasing GSK3B and decreasing CTNNB1 16,39. In this study, overexpression of *MIR133A* regulated the *CDH3*-mediated GSK3B and CTNNB1 signaling pathways (Fig. 3A, B).

The MMP family comprises enzymes that degrade the extracellular matrix. The MMPs play an essential role in morphogenesis and wound healing 40 and are involved in initiating metastasis, invasion, and carcinogenesis from colorectal adenomas 41. Both *MMP1* mRNA and protein are highly expressed in various cancers and play a vital role in initiating tumorigenesis by disrupting the stroma and releasing growth factors. The degradation of the extracellular matrix (ECM) by MMP1 increases cell-ECM interaction, resulting in cell separation from ECM and leading to invasion and metastasis 42. In addition, *MMP2* plays a vital role in disrupting the ECM during metastasis 43. Tumor cells can produce MMP2 or influence host cells to secrete it, as active MMP2 is routinely detected in malignant tumors 44. Overexpression of CDH3 increases MMP activity, which is accountable for cleavage and shedding 45. Our results suggest that MMP1 expression is downregulated by both *MIR133A* and *siCDH3* transfection in CRC cells (Fig. 5A). However, MMP2 expression was only downregulated by *MIR133A* transfection and not by *siCDH3* transfection (Fig. 5B). These data indicate that *MIR133A* regulates CDH3-dependent MMP1 expression, while MMP2 expression is not CDH3-dependent in CRC cells.

Likewise, tumor formation is influenced by the balance between cell growth and apoptosis. If apoptosis is reduced, cell proliferation may become uncontrollable, which leads to cancer 46. Hence, reducing cell proliferation and inducing apoptosis may be an excellent approach for cancer treatment. This study linked cell apoptosis with various genes, like *BCL2, BAX, BIRC5, CASP* family, *RIP3,* and* MLKL*. For example, BCL2 is abnormally expressed in CRC and linked to tumor growth and development, while BAX is a BCL2 family protein and regulates cell development and apoptosis. The expression of BAX is reduced in colon cancer, which is associated with the advancement of cancer 47, 48. Similarly, increased BIRC5 expression prevents apoptosis through inhibition of CASP3 and CASP7 and is also involved in chemoresistance and angiogenesis in CRC. BIRC5 is also directly correlated with anti-apoptotic proteins TACE and MCL1 and inversely correlated with the pro-apoptotic gene BAX 49. In our study, increased BAX and decreased BCL2 and BIRC5 expression were detected in both *MIR133A-*and *siCDH3*-transfected CRC cells (Fig. 4A, B). Associations between CASP3 and CASP8 expression and tumorigenesis and poor prognosis of CRC have been reported 50. Here, our results showed that CASP9, CASP8, and CASP3 are increased by both *MIR133A* and *siCDH3* transfection in CRC cells (Fig. 4A, B). We also assessed the expression of the necroptosis markers RIP3 and MLKL and found that *MIR133A* and *siCDH3* transfection upregulated the expression of MLKL, whereas there was no change in RIP3 (Fig. 4C, D). To further validate the effects on apoptosis, FACS analysis was done, which indicated that transfection of *MIR133A* and *siCDH3* increased apoptosis in CRC cell lines (Fig. 6D). Here, our results indicate that CASP9, CASP8, and CASP3 expression is increased by *MIR133A* transfection in CRC cells (Fig. 4A, B), leading to increased apoptosis (Fig. 6D). Likewise, a study showed that MIR133A is associated with G0/G1 phase arrest and regulates the upregulation of the G1 phase regulator 51.

In addition, colony formation, migration, and MTT assays showed decreased colony formation, reduced migration rate, and diminished cell viability of CRC cell line after transfection with *MIR133A* and *siCDH3* (Fig. 6A, B, C). These results suggest that the reduction of cell viability and impaired migration of CRC cell lines were due to increased apoptosis following *MIR133A* transfection, which was also seen in western blot analyses, as the expression of apoptotic proteins like BAX, CASP9, CASP8, and CASP3 was increased, while that of BCL2 and BIRC5 was reduced (Fig 4). Overall, these data indicate that *MIR133A* regulates apoptosis in the human CRC cell line. Similarly, the regulation of MIR133A in cell migration, colony formation, and invasion is reported by various other studies. MIR133A suppresses cell proliferation viability, migration, and invasion by regulating AQP1 and eIF4A1 and inhibits cell proliferation by targeting SENP1 52,53,54.

## Conclusion

**Figure 8:**
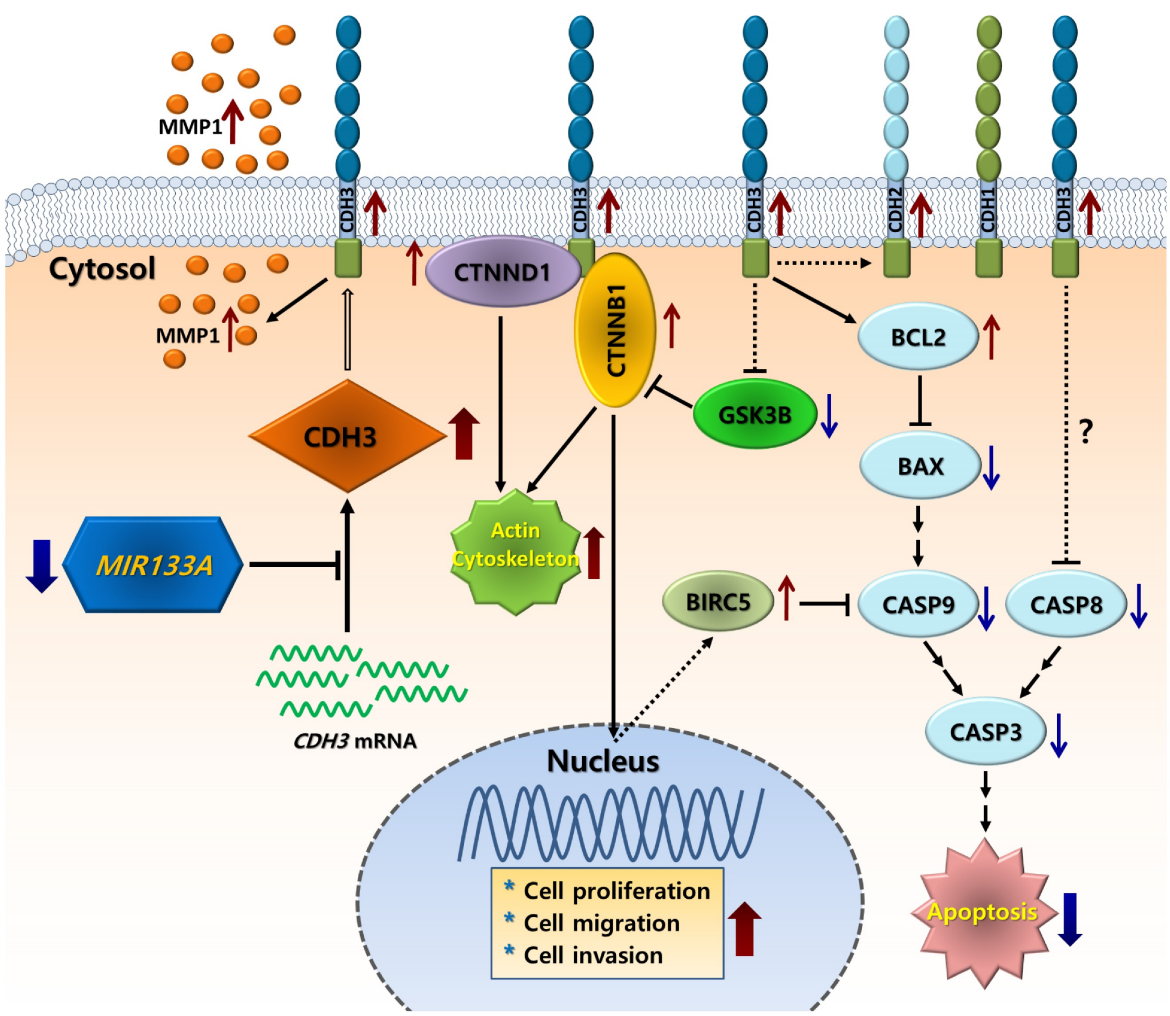
Schematic diagram of the putative mechanism of *MIR133A* in CRC Reduction of *MIR133A* expression in CRC cells or tissues leads to upregulation of cellular CDH3 expression. Upregulation of CDH3 directly or indirectly activates downstream or associated pathways, such as CTNNB1, CTNND1, GSK3B, MMP1, and apoptosis pathways, resulting in increased cell proliferation, cell migration, and cell invasion.

In conclusion, we showed that *CDH3* is a direct target of *MIR133A*, and *CDH3* is overexpressed in CRC tissues in comparison with their respective normal tissues. Expression of both *CDH3* mRNA and protein was significantly downregulated in CRC cells upon transfection with *MIR133A.* The silencing of *CDH3* in CRC cell lines by *siCDH3* produced similar results. Furthermore, our molecular and functional studies suggest that *MIR133A* regulates *CDH3* to induce apoptosis and reduce cell viability, migration, and colony formation in CRC cell lines. Our results suggest that the reduction of *MIR133A* during human CRC progression upregulates *CDH3* expression. The upregulation of intracellular CDH3 may, in turn, affect downstream or associated signal pathways to upregulate cell proliferation, cell migration, and colony formation, as seen in our experiments, while apoptosis was downregulated in CRC cells (Fig. 8). Although we did not demonstrate these effects *in vivo*, *MIR133A* produces a potent antitumor effect *in vitro*. Our results suggest that *MIR133A* regulates *CDH3* in human CRC; therefore, *MIR133A* may be a therapeutic target in human CRC, which needs to be examined further.

## Supplementary Material

Supplementary figure and table.

## Abbreviations

3' UTR: three prime untranslated region
BAX: BCL2 associated X
BCL2: BCL2 apoptosis regulator
BIRC5: baculoviral IAP repeat-containing 5
CASP3: caspase 3
CASP8: caspase 8
CASP9: caspase 9
CDH1: epithelial cadherin
CDH2: neural cadherin
CDH3: cadherin 3
CRC: colorectal carcinoma
CTNNB1: catenin beta 1
CTNND1: catenin delta 1
EMT: epithelial-mesenchymal transition
GSK3β: glycogen synthase kinase 3 beta
IBD: inflammatory bowel disease
IHC: immunohistochemistry
MIR133A: microRNA 133A
MLKL: mixed lineage kinase domain-like pseudokinase
MMP1: matrix metalloproteinase-1
MMP2: matrix metalloproteinase-2
qRT-PCR: quantitative Real time polymerase chain reaction
RIP3: receptor-interacting serine/threonine kinase 3
siCDH3: small interfering cadherin-3
VIM: vimentin
