# Supplementary material for: MicroRNA 133A Regulates Cell Proliferation, Cell Migration, and Apoptosis in Colorectal Cancer by Suppressing CDH3 Expression
Source: J Cancer. 2023 Apr 9;14(6):881–94. doi: 10.7150/jca.82916 (PMC10158507; doi:10.7150/jca.82916)
Supplement: Supplementary file 1 — Supplementary figure and table. [file jcav14p0881s1.pdf]

Supplementary Figure

Fig. S1

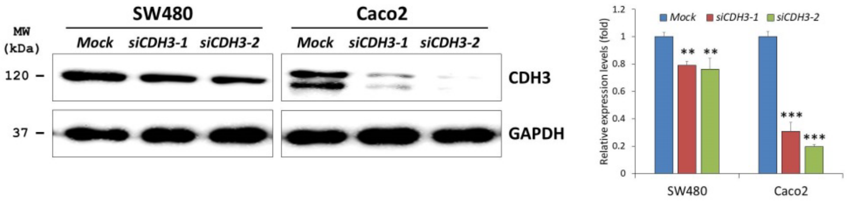

Figure S1. Western blot analyses of *siCDH3*-1 and *siCDH3*-2 in SW480 or Caco2 cell line

In this experiment, we used two siRNA to silence the *CDH3* gene. As shown in the figure, *siCDH3*-2 produced more complete silencing of the *CDH3* gene than *siCDH3*-1. Hence, we used *siCDH3*-2 in further experiments. Differences were considered statistically significant compared with control (** $P < 0.01$, *** $P < 0.001$).

Supplement Table

Table 1. Primer sequences used for PCR amplification and luciferase assay in this study

Applications	Primers	Primer sequence (5' → 3')
qRT-PCR	CDH3-F	GGCAGACATGTACGGTGGCGG
	CDH3-R	GCCAAGTTGCTACGGCCACTTCC
	GAPDH-F	TCACCATCTTCCAGGAGCGAGA
	GAPDH-R	TCACTGGCATGGCCTTCCGTG
Luciferase assay	CDH3-WF	GCCTGCAGGGCTCGTCAGGCCACAGAGC
	CDH3-WR	GCTCTGTGGCCTGACGAGCCCTGCAGGC
	CDH3-MF	GCAGAGCTCCCTCACCTCCTCCGCCTCC
	CDH3-MR	CGTCTCGAGCGTCAGACTCATAGCCTGTCTCC
